# The Nile from the Point of View of Health

**Published:** 1921-06-25

**Authors:** 


### THE NILE FROM THE POINT OF VIEW OF HEALTH.

THREE winters spent as medical officer on Cook's Nile steamers have convinced the writer that medical men do not sufficiently realise that, therapeutically speaking, Egypt may mean various things. A doctor sends a patient to Egypt because he is suffering from some disease or ailment likely to be benefited by a warm and dry climate. Good and well. But Egypt has varying degrees of warmth and dryness to offer. Between Alexandria and Assouan there are nearly 700 miles, and as much difference in climate as between London and Cannes. Alexandria has a marine, Assouan a desert, climate. Cairo is a city, Luxor is a country village. We have met invalids in Cairo who have been sent to Egypt for their health, and who have thought it a matter of complete indifference whether they spent the winter months in Cairo or Alexandria, or Helouan or Luxor. It is not a matter of indifference at all : it may indeed be a matter of vital importance. A lung patient can hardly do much worse than spend a winter in Cairo, dividing his time between dancing at "Shepheard's" and shopping in dusty and dirty bazaars. Patients should accordingly be instructed exactly where to winter and how to live.

To discuss the whole subject here would be impossible, but one may at least briefly point out a few facts with regard to a trip on the Nile.

A trip from Cairo to Assouan and back takes about three weeks. During the southward journey the weather usually grows gradually warmer, and during the northward journey gradually cooler; but the temperature varies greatly with the direction of the wind. The wind is usually from the north, and the journey down the Nile in the face of the wind may be quite cool. The humidity of the air, though low, is probably slightly more than on land, but it also varies with the direction of the wind, and when a "Khamsin" wind is blowing it becomes very low. The dryness of the air renders the difference between sun temperature and shade temperature very marked, and causes a rapid fall in temperature at sunset. One may say, in a general way, that the climate is ideal. At Cairo the temperature may vary between 50° F. and 80° F., with a mean of 60° F.; at Assouan, between 60° F. and 90° F., with a mean of 66° F. The relative humidity at Cairo will be about 50 and at Assouan about 30. The sun shines from sunrise to sunset, and there is no rain. The oscillations of temperature, it is true, are considerable—sometimes as much as 36° F. in the twenty-four hours—and may cause physiological disturbances, but on the whole the oscillations are bracing and stimulating. A more delightful and healthful holiday than a trip from Cairo to Assouan and back there could not be. Cook's steamers are the last word in comfort and luxury; the food is excellent, the scenery is beautiful, and temples, palaces, and tombs afford continual intellectual interest. Yet the trip should not be prescribed indiscriminately, and the prescription should be accompanied by instructions. The trip will be beneficial for patients with weak chests and throats only under certain conditions, and the main condition is that they keep on the boat and abjure all excursions. On the boat they are free from dust and germs; but if they go in cavalcade to visit tombs and temples, they not only face fatigue but clouds of dust, with consequent irritation of their respiratory mucous membranes. The fatigue and the dust together will undo most of the good of the trip. Nasal and pharyngeal catarrhs are frequent sequelæ of donkey excursions. And still more frequent sequelæ are they of visits to dusty and dirty bazaars. The catarrhs are usually attributed to "chills" consequent on sudden changes of temperature, but they are not due to "chills" —they are due simply to dust, and though the desert dust may be aseptic, the bazaar dust is probably full of germs. If a patient with vulnerable respiratory mucous membranes will live on the boat and eschew shore excursions he will undoubtedly derive benefit from the trip.

Cases of neurasthenia and mental depression also derive benefit from a Nile trip, and in such cases the excursions across the desert will be part of the treatment. The restful, peaceful life on the boat, as it glides along, constitutes a rest-cure under ideal conditions, and the exhilarating desert air breathed from the back of a galloping donkey makes depression almost impossible.

Cases of rheumatism, rheumatoid arthritis, and Bright's disease invariably improve in the dry stimulating air, but such cases when they reach Assouan will do well to make a prolonged stay there, since the relative humidity there is less than at any other tourist resort in Egypt. Cases of Bright's disease should certainly not return to England till the middle of May, for after a sojourn in the dry and sunny air of Egypt cold and damp will suddenly double the work of the kidneys and be doubly deleterious.

